# Systematic Review and Quality Evaluation Using ARRIVE 2.0 Guidelines on Animal Models Used for Periosteal Distraction Osteogenesis

**DOI:** 10.3390/ani11051233

**Published:** 2021-04-24

**Authors:** Mario García-González, Fernando Muñoz, Antonio González-Cantalapiedra, Mónica López-Peña, Nikola Saulacic

**Affiliations:** 1 Department of Veterinary Clinical Sciences, Faculty of Veterinary, Universidade de Santiago de Compostela, 27002 Lugo, Spain; fernandom.munoz@usc.es (F.M.); antonio.cantalapiedra@usc.es (A.G.-C.); monica.lopez@usc.es (M.L.-P.); 2 Department of Cranio-Maxillofacial Surgery, Faculty of Medicine, University of Bern, 3010 Bern, Switzerland; nikola.saulacic@insel.ch

**Keywords:** animal models, periosteal distraction osteogenesis, osteogenic distraction, bone regeneration, systematic review

## Abstract

**Simple Summary:**

Periosteal distraction osteogenesis (PDO) is a promising new technique for bone regeneration, as it avoids some of the complications that other techniques present. In this review, were examined the animal models used in preclinical studies carried out so far, as well as the quality of the studies using the ARRIVE guidelines (Animal Research: Reporting of In Vivo Experiments). The models that have shown the best results in terms of handling and fewer complications are the rabbit and the rat. The minipig is not recommended due to its difficult oral hygiene and handling. The quality of the studies has increased since the implementation of the ARRIVE guidelines in 2010. Future studies shall be improved in terms of transparency, comparability, and reproducibility.

**Abstract:**

The objective of this systematic review was to synthesize all the preclinical studies carried out in periosteal distraction osteogenesis (PDO) in order to evaluate the quality using the ARRIVE guidelines. The animal models used, and the influence of the complications, were analysed in order to establish the most appropriate models for this technique. The PRISMA statements have been followed. Bibliographic sources have been consulted manually by two reviewers. Risk of bias was evaluated using the SYRCLE tool for animal studies, and the quality of the studies with the ARRIVE 2.0 guidelines. The selection criteria established by expert researchers were applied to decide which studies should be included in the review, that resulted in twenty-four studies. Only one achieved the maximum score according to the ARRIVE 2.0 guidelines. The rabbit as an animal model has presented good results in PDO, both for calvaria and jaw. Rats have shown good results for PDO in calvaria. The minipig should not be recommended as an animal model in PDO. Despite the increase in the quality of the studies since the implementation of the ARRIVE 2.0 guidelines, it would be necessary to improve the quality of the studies to facilitate the transparency, comparison, and reproducibility of future works.

## 1. Introduction

Nowadays, bone regeneration of extended bone deficiencies is one of the most important challenges in reconstructive surgery. The most common techniques used for the treatment of bone defects are the autogenous bone grafting, distraction osteogenesis (DO) and guided bone regeneration (GBR). Although bone grafts are still the “gold standard”, the technique may represent morbidity, a limited quantity of the donor site, pain, and complications such as osteonecrosis or bone resorption. DO consists of the gradual separation of two bone segments after an osteotomy or corticotomy. It can generate enough bone, but it is invasive, patient’s compliance is necessary, and the duration of treatment is long. GBR consists of covering the bone filler material with a membrane, which prevents the invasion of soft tissues in the augmented site and maintains the blood clot. Its main disadvantage is the quality of the regenerated bone and limited capacity of vertical bone augmentation [1]. 

A new technique has emerged in recent years, entitled periosteal distraction osteogenesis (PDO). PDO is considered as the combination of the DO and GBR, since its objective is to create a space between the periosteum and the bone surface by expanding the periosteum, together with the skin and the muscle. PDO does not require osteotomy compared to DO. Additionally, the morbidity of the donor site may be avoided since bone harvesting is not required and does not present immune complications. The main limitation is that the technique requires highly qualified professionals [2].

The periosteum plays an important role in osteogenic distraction due to its highly vascularised inner cambium layer. It contains many stem cells, which have the capacity for osteogenesis [1,3].

The periosteal distraction technique can be used for the reconstruction of bony defects in the forehead region in the craniomaxillofacial surgery or in neurosurgery; solve problems of bone deficit in the alveolar bone in oral implantology; or for volume deficits that sometimes occur in vertical distraction or bone grafts [4,5].

PDO comprises the following stages: (A) surgery, (B) latency, (C) distraction, and (D) consolidation [3]. The first authors that studied the PDO technique in bone formation performed in the mandibular region were Schmidt et al. [6].

Since then, several preclinical studies have been conducted to study the bone formation by PDO. However, a consensus has not been reached on the ideal protocol to perform the intervention yet, since there is a great diversity of variables, such as the animal models used, devices, anatomical sites or variations in the surgical technique and parameters of distraction (in terms of latency period, frequency and activation of the device and period of consolidation) [3].

The Animals in Research Reporting In Vivo Experiments (ARRIVE) guidelines is a checklist intended to provide transparent and accurate reports of animal studies. It has been developed in 2010 and updated in 2020 (ARRIVE 2.0) to solve the reproducibility problem in animal research. Since then, many journals have made its use mandatory when reporting original research [7,8,9].

At the present time, there have been no systematic or literature reviews on preclinical studies in PDO focused on the animal model and their complications. Furthermore, it has not been evaluated whether these studies have been developed following the ARRIVE guidelines.

The aim of this systematic review is synthesizing all the preclinical studies carried out in PDO using the ARRIVE 2.0 guidelines, the animal models used, and the complications encountered were assessed, in order to evaluate their quality, and establish the most appropriate models for this research.

## 2. Materials and Methods

This systematic review follows the Preferred Reporting Items for Systematic Reviews and Meta-Analyses (PRISMA) statements [10] and the animal systematic review protocol made by Vries et al., 2015 [11]. The studies have been collected from the following health science databases: PubMed, Web of Science (WOS) and Scopus (limiting the search until December 2020). This collection was made manually during the month of December 2020 by two reviewers (MG-G and FM).

### 2.1. Search Strategy

The search strategy used the following clauses:

- Animal model AND preclinical studies AND (periosteal distraction osteogenesis OR osteogenesis distraction OR periosteum).
- Periosteal distraction osteogenesis AND (bone augmentation OR bone regeneration).
- Animal AND periosteal distraction.

### 2.2. PICO Methodology

Animal models (P = patients), preclinical studies (I = intervention), of different species (C = comparison), used for PDO (O = result). PICO question: What is the most appropriate animal model to use in PDO preclinical procedures? 

### 2.3. Inclusion Criteria.

1. Experimental studies of PDO aimed at bone regeneration with animals used as biological models.
2. Studies indexed in JCR (Journal Citation Reports).
3. Articles in English.

### 2.4. Quality Assessment and Risk of Bias

The ARRIVE 2.0 guidelines for reporting animal research were used to evaluate the quality of the studies [9]. In order to evaluate the 21 items, it was indicated with “reported (= 2 points)” if the publication complied with all subitems, “not reported (= 0 points)” if it did not, and “unclear (= 1 point)” if the details were not provided for all subitems.

In this way, a pre-defined quality coefficient (0.8–1 Excellent, 0.5–0.8 Average, <0.5 Poor) [12,13] were applied to each study, calculated as the sum of all the points obtained for each study, and divided by 42 (the maximum possible points per study). In order to evaluate the items, the percentage of reported, not reported or unclear items were be calculated as well as the coefficient (the maximum possible for each item is 48 points).

The items evaluated according to ARRIVE 2.0 were divided in two groups. On the one hand, the essential 10: (1) study design, (2) sample size, (3) inclusion and exclusion criteria, (4) randomisation, (5) blinding, (6) outcome measures, (7) statistical methods, (8) experimental animal, (9) experimental procedures, (10) results. On the other hand, the recommended set: (11) abstract, (12) background, (13) objectives, (14) ethical statement, (15) housing and husbandry, (16) animal care and monitoring, (17) interpretation/scientific implications, (18) generalisability/translation, (19) protocol registration, (20) data access, (21) declaration of interests.

### 2.5. Risk of Bias 

Risk of bias was assessed using the Systematic Review Centre for Laboratory animal Experimentation (SYRCLE) tool for animal studies [14]. The risk of bias tool is made up of 10 items with specific signalling questions. In order to assign an assessment of high, low or unclear risk of bias to each item, it was indicated as “Yes” when the risk of bias is low, “no” indicated high risk of bias and “unclear” indicated that the details are insufficient to adequately assess the risk of bias. Items had unclear risk of bias if one or more sub-questions were partly satisfied, and high risk of bias if one or more sub-questions were not met.

The quality and risk of bias of the studies was assessed independently and in duplicate by two reviewers (M.G.-G. and F.M.) and the level of agreement between both was calculated using Kappa statistics.

### 2.6. Analysis and Extraction of Parameters of Interest

The studies were valued according to the following items: animal model (species, breed, sex, age, weight and patient number), device (type of distractor, number of devices used and anatomical region), distraction phases (latency, activation (distraction) and consolidation), rate and frequency of distraction, evaluation methods, results (qualitative histology, bone mineral density (BMD), bone volume (BV), ratio bone volume/tissue volume (BV/TV), new bone area (NBA), % of new bone (% NB), trabecular thickness (Tb.Th), height and width gain) and complications observed.

Regarding the studies in which complications were reported, they were classified as minor and major [15]. In addition, the phase in which they were observed was detailed. 

### 2.7. Statistical Analysis

Descriptive analysis (mean ± standard deviation; median, if applicable) was calculated. Inter-reviewer agreement was quantified using kappa (k) statistics for the quality evaluation of the studies. Statistical analysis was performed using SigmaPlot^®^ 12.5 for Windows (Systat Software Inc., San José, CA, USA).

## 3. Results

**Figure 1:**
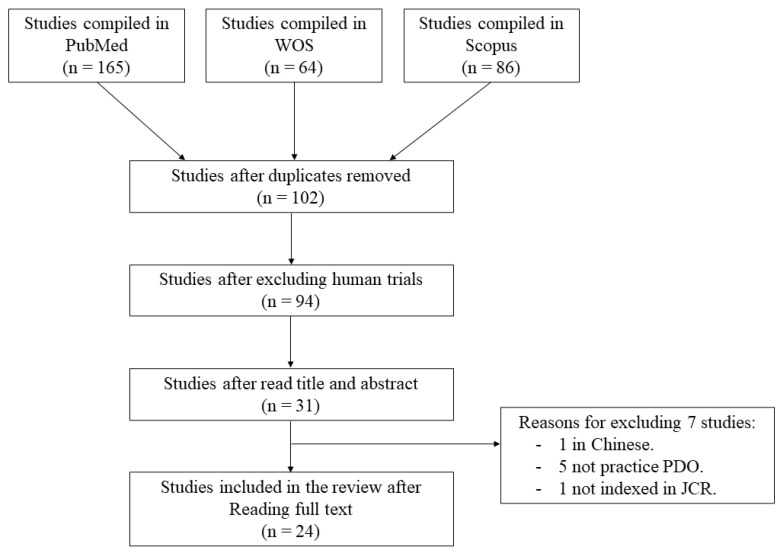
Flow chart of the selected studies.

After the initial search, 315 articles were generated. The flow chart of the selected articles is shown in the Figure 1. After the exclusion of duplicates and studies in humans, a total of 94 studies remained. Based on titles and abstracts, 31 studies were selected. Finally, after reading full text and applying study criteria, 24 studies [2,4,5,6,16,17,18,19,20,21,22,23,24,25,26,27,28,29,30,31,32] were included in the review (inter-reviewer agreement k = 0.96).

The 24 studies selected involved 481 patients with 482 devices. Fifteen studies were in rabbits (with a total of 327 patients and 309 placed devices), 4 in rats (122 patients and 122 devices), 3 in minipigs (24 patients and 33 devices) and 2 in dogs (8 patients and 18 devices). The most used breed was New Zealand white rabbits, Wistar rats and Gottingen minipigs. The breeds of the dogs were not reported.

All animals used were adults and skeletally mature. The average ages by species were 4.5 months in rabbits, 19 months in dogs and 12.7 in minipigs. The average weight was 14.2 kg in dogs (ranging from 10 to 16 kg), 3 kg in rabbits (ranging from 2.5 to 4.15), 0.33 kg in rats (ranging from 0.3 to 0.4) and 28.2 kg in minipigs (ranging from 20 to 34) (Table 1). All rats were adults, but the exact age was not reported.

### 3.1. Indication and Location for Distraction

In 11 studies (217 patients), the mandible was used for the PDO (9 extraoral and 2 intraoral), and in 13 (264 patients) some region of the skull (4 used the forehead region and 9 the calvaria).

### 3.2. Device Details

A total of six different distraction devices and prototypes were used: U-shaped device (7 studies, 212 patients, 194 devices), titanium plate (7 studies, 134 patients, 144 devices), titanium mesh (6 studies, 81 patients, 90 devices), Biodegradable PLLA mesh (2 studies, 26 patients, 26 devices), hemispherical disc (1 study, 16 patients, 16 devices) and modified hyrax (1 study, 12 patients, 12 devices).

### 3.3. PDO Protocol

A latency period of 7 days was used by most of the studies (15 studies), with a mean of 6.5, ranging from 1 and 14 days. A distraction period of 10 days was the most common (11 studies), with an average of 10.3, ranging from 5 to 22 days. The mean distraction rate was 0.52 (0.1 to 1) mm per day. The distraction frequency varied between 1 and 2 times per day. The consolidation period average was 34.3 days (4.9 weeks), ranging from 7 to 90 days (1 to 12.86 weeks). Table 2 shows in detail the devices and protocols used.

### 3.4. Evaluation Methods and Results

The evaluation methods (Table 1) used were qualitative histology (22 studies), micro-computed tomography (13 studies), histomorphometry (9 studies), radiology (5 studies), immunohistochemistry (1 study) and photo-densitometry (1 study). Quantitative results were evaluated mainly by evaluating BV (11 studies), BMD (6 studies), NBA (5 studies), height gain (5 studies), BV/TV (4 studies), % NB (2 studies), Tb.Th (1 study) and width gain (1 study). Supplementary Table S2 shows in detail the results of each study.

### 3.5. Complications and Treatment

Complications were encountered in 12 studies. Nine studies declared no complications and 3 of them provided no information.

Thirty-five patients (7.3% of the total) evidenced complications; in 28 of them they were major (5.8% of the total) and in 7 they were minor (1.4% of the total). By order of frequency, the major complications were severe infection (14 patients, 40% out of complications), device lost (6 patients, 17.13%), dehiscence (4 patients, 11.43%), post-operative death (2 patients, 5.72%), moderate device displacement (1 patient, 2.86%) and body weight loss > 15% (1 patient, 2.86%). Using the same criteria, the minor complications were slight device displacement (5 patients, 14.28% out of the complications) and slight infection (5.72 patient, 6%).

Analysing the complications by species, the rabbit was the species that showed more complications, since 16 animals had major complications (45% out of complications) and 4 minor complications (11.43%), followed by the rat, showing 7 patients with major complications (20%). Four dogs experienced major complications (11.43%). Finally, 1 minipig had major complications (2.86%) and 3 minor complications (8.57%).

Analysing complications by type of device, the titanium plate presented 21 patients with major complications (60% out of complications; 12 rabbits, 5 rats and 4 dogs), the titanium mesh presented 2 major complications (5.71%; 1 in rabbit and 1 in pig) and 3 minor complications (8.58%; 3 in pigs), U-shaped device presented 3 major complications (8.58%; 3 rabbits), hemispherical-disc showed 2 major complications (5.71%; 2 rats), PLLA mesh 2 minor complications (5.71%; 2 rabbits) and lastly modified hyrax 2 minor complications (5.71%; 2 rabbits).

In 1.46% (4 pigs and 3 rabbits) of the total patients, the devices were affected due to loss and displacement.

One of the studies was a general failure [28], due to dehiscence, displacement and loss of devices and severe infection in all patients. Sixteen of the 38 patients (4 dogs and 12 rabbits) belong to this study where a titanium plate was used. 

Table 3 shows in detail the complications by study.

### 3.6. Quality Assessment of Selected Studies

**Figure 2:**
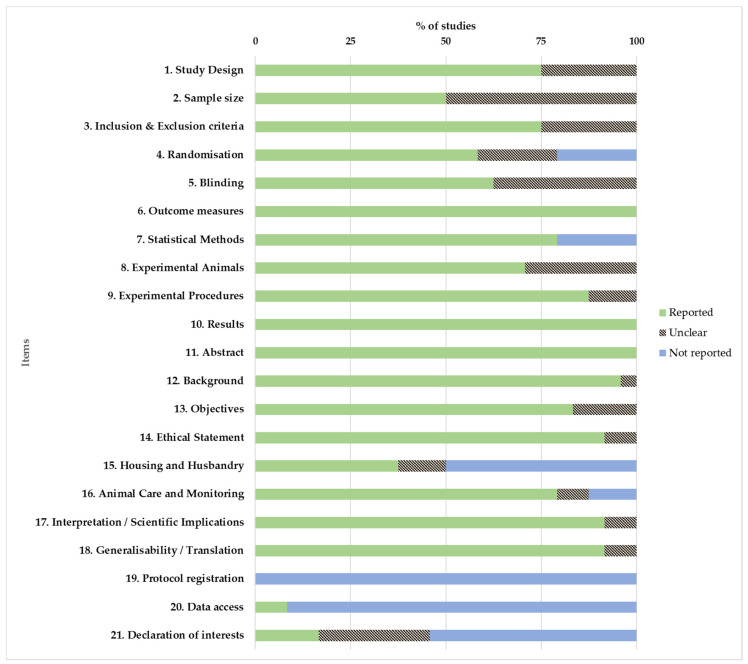
Quality assessments of the studies according to ARRIVE 2.0 guidelines. Values are expressed by %.

The quality coefficients of the studies are shown in Table 4. The percentage frequencies of each item are shown in Figure 2. Regarding the studies, 9 were rated as excellent (coefficients between 0.8 and 1), and 15 as average (coefficients between 0.5 and 0.8). None of the studies achieved the maximum coefficient of 1. In relation to the items, 14 were scored as excellent: (1) study design, (3) inclusion and exclusion criteria, (5) blinding, (6) outcome measures, (8) experimental animals, (9) experimental procedure, (10) results, (11) abstract, (12) background, (13) objectives, (14) ethical statement, (16) animal care and monitoring, (17) interpretation/scientific implications, (18) generalisability/translation. Three items were scored as average: (2) sample size, (4) randomisation, (7) statistical methods. Lastly, 4 items were scored as poor (coefficients between 0 and 0.5): (14) housing and husbandry, (19) protocol registration (20) data access, (21) declaration of interest.

### 3.7. Risk of Bias in Studies

**Figure 3:**
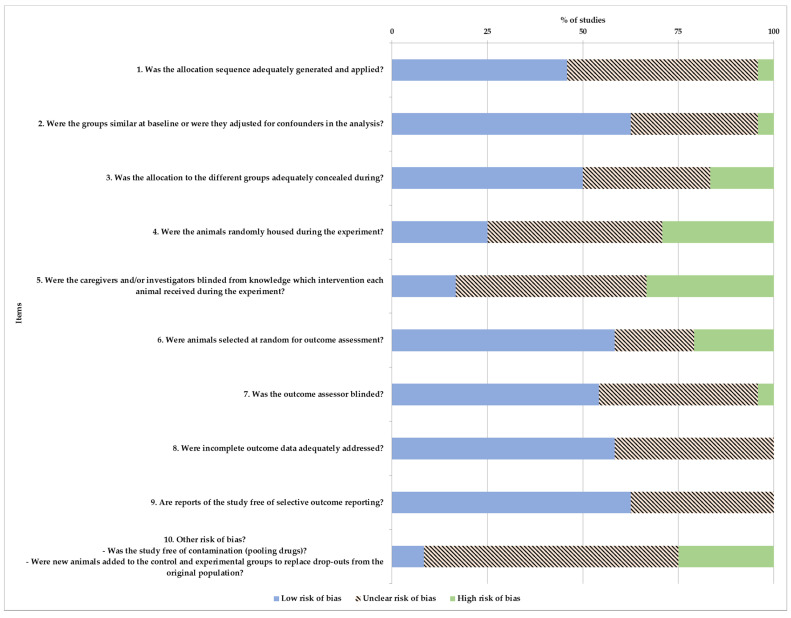
Risk of bias distribution according to Systematic Review Centre for Laboratory Animal Experimentation (SYRCLE) tool. Values are expressed by %.

Detailed results are listed in Figure 3. Mostly, the items were assessed as low risk of bias. The lower risk of bias was assigned at items’ (2) baseline characteristics, (3) allocation concealment, (6) random outcome assessment, (7) blinding of outcome assessor, (8) incomplete outcome data and (9) selective outcome reporting, with frequencies of 62.5%, 50%, 58.34%, 54.17%, 58.34% and 62.5%. The higher risk of bias was observed at items (4) random housing, (5) blinding of caregivers and/or investigators and (10) other sources of bias, with frequencies of 29.16%, 33.34% and 25%.

## 4. Discussion

In the present review, the quality of the studies on animal models used in PDO has been assessed according to the ARRIVE 2.0 guidelines. A total of 24 studies on PDO have been selected, according to the previously established inclusion criteria.

### 4.1. Animal Models and Complications

Despite being a relatively new technique, a large variety of biological models used for its research have been found (rabbits, rats, dogs and minipigs).

A lower percentage of complications has been found compared with VAOD (vertical alveolar osteogenesis distraction) technique (7.3 vs. 9.95%) [35]. Furthermore, the device loss rate compared to VAOD is also lower than in PDO (1.46 vs. 3.31%). This may be because periosteal distraction does not require an osteotomy and can avoid possible complications such as fractures, deviation of the inclination vector or fragment sequestration [3,36]. The rabbit has presented the highest number of complications (16 animals; 45% complications), but it has been the most used species (327 of 481 patients). In addition, 12 animals that presented complications (34.3% complications) belong to a single study that failed due to severe infections [28].

The rabbit model was sometimes chosen because the size of its jaw is adjusted to the size of the device, and its ability to return to normal dietary habits quickly. However, on the other hand, the different osteogenic response compared to humans should be considered [6,34]. Besides, their clinical relevance is limited regarding human patients, given the discrepancy in mandibular size, morphology, and function of their counterparts [37].It has been also observed that the animal stops eating due to pain caused by the device in the oral cavity. Given the absence of chewing stimulus, bone maturation can be affected [27,30]. 

Only two studies have been reported in which the PDO technique has been implemented in dogs. While in one of them there were no complications [31], in the other a high rate of complications was reported [28]. Thus, a conclusion on the suitability of the Mongrel dog as an animal model cannot be made, corresponding to the use in VAOD [35]. Future studies should investigate the dog as an animal model in PDO.

In pigs, one major and three minor complications have been reported. However, the difficulty of handling, and oral hygiene maintenance, as well as a high incidence of infection [38] have to be considered. Minipig is not a recommendable model for PDO in the absence of further studies, especially given the frequent complication rates and high costs previously observed in VAOD [35].

Rats have shown good results regarding their use in calvarial PDO studies. They are small and easy to handle, with large samples sizes often used. As in rabbits, the clinical relevance in humans should be considered. 

In order to reduce the number of animals, some authors recommend the use of larger animals because they can support various devices, including control [37]. However, the ethical implications arising from its use should be carefully considered and used only when the devices are in the final stages of investigation.

It has been shown that the age of the patient may influence the results [39]. In this study, as in the previous study carried out in VAOD [35], all the animals included were adults; the results were thus not influenced by the age of the patient.

### 4.2. Protocol

In relation to the protocol, there are still some discrepancies about the ideal in PDO. As in VAOD, the phases have been the same (surgery, latency, distraction, and consolidation). In this review, most studies have used a 7-day latency (the same in VAOD), with a mean of 6.5 (6.13 in VAOD). Previous studies in humans on conventional DO have not found differences using different latency periods [40]. Even so, a latency period of 4–7 days is recommended to avoid premature bone exposure, especially in animal models [41]. Regarding the distraction period, most studies used 10 days, with a mean of 10.3 (9.15 in VAOD). Frequency and rate of distraction were one or two times per day, and a mean of 0.52 mm per day (0.8 in VAOD). More than two times per day can damage the procedure, and more than 1 mm of distraction per day is not recommended [42]. Finally, in the consolidation period, a mean of 4.9 weeks (ranging from 1 to 12.86 weeks) was reported (9.93 in VAOD). A minimum of 10 weeks is recommended to observe the complete consolidation in VAOD in humans [42], but shorter consolidation periods in animals can be explained because of the faster metabolism compared to humans.

### 4.3. Strengths and Limitations of the Data

As well as evaluating the animal models used in PDO, the present review also sets out to identify the shortcomings of the reported preclinical studies. According to the ARRIVE standards for animal experimentation with animal models [7,8], an increase in the quality of studies has been observed since its imposition. However, most of the studies have not followed all the recommendations proposed by these guidelines. This may be because not all the journals which published the studies included in this review suggested or obliged the use of the ARRIVE 2.0 guidelines.

In this review, the maximum score according to the ARRIVE 2.0 guidelines was 36 points. Even though nine studies have obtained a rank of excellent, none of them achieved the maximum score. Analysing the categories, only three were reported in all the studies: (6) outcome measures, (10) results, and (11) abstract. The categories with the lowest coefficients were (19) protocol registration, (20) data access, and (21) declaration of interest. Most of the studies were rated as excellent (coefficients between 0.8 and 1) as they consistently reported the criteria. It should be noted that a high number of studies did not adequately justify the use of the animal model. These values agree with the results from systematic reviews obtained by Delgado-Ruiz et al. [12], who evaluated the critical size defects in bone regeneration experiments in rabbit calvaria, and with Schwarz et al. [13] who evaluated the treatment of peri-implantitis.

The present study has some limitations. When the included studies are categorised according to the ARRIVE 2.0 guidelines, the same weight is given for the ten essential items as for the other eleven.

If an experimental study is not designed to produce robust results, and publications are not reported in enough detail, the research resources and the animals are wasted. According to Kilkenny et al., [7,43] when addressing the quality of information from experimental animal studies, the lack of a comprehensive and systematic approach in method description can lead to confusing reports, with both ethical and economic implications. Animal experiments should be correctly designed, appropriately analysed, and transparently reported to the scientific community in order to increase their reliability and scientific validity, maximizing the knowledge gained from each investigation. For this reason, the ARRIVE 2.0 guidelines have been designed.

All studies included in this review had bioethical approval by a competent committee. Despite this, two studies were classified in item 14 (“Ethical Statement”) as “unclear” because they did not report the name of the ethics committee or institution that gave them authorisation to carry out the study.

The risk of bias of the studies conducted in this review was a low risk of bias, although four categories resulted in an unclear risk. In bone regeneration studies with animals it is especially important to report the randomisation of the devices and protocols in the methodology, since the quality of regenerated bone can vary within various sites in an animal [12,44].

In accordance with all above, it would be necessary to improve the quality of animal preclinical studies in terms of essential details, to facilitate comparison and reproduction in future works.

## 5. Conclusions

The PDO technique presents fewer complications and a lower device loss rate in preclinical studies compared to VAOD.

The rabbit as animal model has presented good results for PDO, both for calvaria and jaw. Rats have shown good results in their use as a model for PDO in the calvaria. Given its difficult handling and difficult oral hygiene, the minipig would not be recommended as an animal model in PDO.

Despite of the improvement since the ARRIVE guidelines have been implemented, it would be necessary to enhance the quality of the studies to facilitate the transparency, comparison, and reproducibility of future works.

## Figures and Tables

**Table 1:** Characteristics of the animal model and evaluation methods used by each study.

Author	Year	Animal	Breed	Age	Sex	Weight (kg)	N° Patients	Evaluation Method
Schmidt et al. [6]	2002	Rabbit	New Zealand	Adult	Male	3.9 ± 0.39	10	Histologic and histomorphometric
Sencimen et al. [27]	2007	Rabbit	New Zealand	Adult	Male	3.55 ± 0.65	36	Histologic and histomorphometric
Estrada et al. [28]	2007	Dog	-	20 months-old	-	16	4	Radiographic and histologic
Casap et al. [29]	2008	Rabbit	New Zealand	10 months-old	Male	2.9	10	Micro-CT and histomorphometric
Oda et al. [33]	2009	Rabbit	Japanese	Adult	Male	3.2–3.7	25	Radiographic and histologic
Altuğ et al. [30]	2011	Rabbit	New Zealand	Adult	-	4.15 ± 0.55	36	Histologic and histomorphometric
Bayar et al. [34]	2012	Rabbit	New Zealand	6 months-old	Female	3.6	36	Histologic and histomorphometric
Inoue et al. [31]	2014	Dog	-	1–2 years-old	Female	10 to 15	4	Micro-CT
Suer et al. [32]	2014	Rabbit	New Zealand	Adult	Male	3.7 ± 0.55	24	Radiologic, photodensitometric and histologic
Kahraman et al. [16]	2015	Rabbit	New Zealand	Adult	-	3.05 ± 0.15	20	Radiographic, micro-CT, histologic and histomorphometric
Pripatnanont et al. [17]	2015	Rabbit	New Zealand	Adult	Male	3.5	12	Micro-CT, Histologic and histomorphometric
Kessler et al. [4]	2006	Minipig	Göttingen	2–3 months-old	Female	20–25	6	Micro-CT and histologic
Estrada et al. [28]	2007	Rabbit	New Zealand	4 months-old	-	3.5	12	Radiographic and histologic
Lethaus et al. [18]	2010	Minipig	-	Adult	Female	34 ± 4.8	9	Histologic and micro-CT
Sato et al. [19]	2010	Rabbit	New Zealand	3–4 months-old	Male	3	8	Micro-CT, histologic and inmunohistochemistry
Tudor et al. [2]	2010	Minipig	Gottingen	23 months-old	Female	24 ± 4.8	9	Histologic and micro-CT
Zakaria et al. [20]	2012	Rabbit	Japanese	Adult	Male	2.5–3	8	Histologic and micro-CT
Zakaria et al. [21]	2012	Rabbit	Japanese	1.5 months-old	Male	2.5–3	12	Histologic and micro-CT
Saulacic et al. [22]	2013	Rat	-	-	-	-	16	Histologic and histomorphometric
Saulacic et al. [23]	2013	Rat	Wistar	Adult	Male	0.4	48	Histologic and histomorphometric
Saulacic et al. [24]	2016	Rabbit	New Zealand	Adult	Female	3	60	Histologic and micro-CT
Nakahara et al. [25]	2016	Rat	Wistar	Adult	Male	0.3	28	Histologic and micro-CT
Nakahara et al. [26]	2017	Rat	Wistar	Adult	Male	0.3	30	Histologic and micro-CT
Zhao et al. [5]	2020	Rabbit	New Zealand	1.5–2 months-old	Male	2.5–3	18	Histologic and micro-CT

**Table 2:** Characteristics of the protocols and devices used in each study.

Author	Year	Animal	Breed	Distractor	N° Devices	Anatomical Region	Latency Period (Days)	Distraction Period (Days)	Frecuency/Rate	Consolidation Period (Days or Weeks)
Schmidt et al. [6]	2002	Rabbit	New Zealand	U-shaped body, Synthes Maxillofacial, Paoli, Pa	10	Lateral surface of the mandible (E)	7	15	7 mm over 15 days	4, 5, 6, 8 w
Sencimen et al. [27]	2007	Rabbit	New Zealand	U-shaped device	18	Lateral surface of the mandible (E)	7	10	0.25 mm/12 h	15, 30, 60 d
Estrada et al. [28]	2007	Dog	-	Titanium plate, Tracper TM Serf. Décines, France	12	Intraoral in the four quadrants (I)	10	22	0.22 mm/d	90 d
Casap et al. [29]	2008	Rabbit	New Zealand	U-shaped device	10	Mandible (E)	14	7	1 mm/d	60 d
Oda et al. [33]	2009	Rabbit	Japanese	Titanium mesh, M-TAM, Stryker Leibinger, Kalamazoo, MI	25	Mandible (E)	7	8	0.5 mm/d	4 and 8 w
Altuğ et al. [30]	2011	Rabbit	New Zealand	U-Shaped device	36	Mandible (E)	1 or 7	10	0.25 mm/12 h	15, 30, 60 d
Bayar et al. [34]	2012	Rabbit	New Zealand	U-shaped device	36	Mandibular corpus (E)	7	10	0.25 mm/12 h	15, 30, 60 d
Inoue et al. [31]	2014	Dog	-	Titanium plate	6	Mandible PM1-M1 (I)	24	6	0.5 mm/d	8 w
Suer et al. [32]	2014	Rabbit	New Zealand	U-shaped device	24	Lateral surface of the mandible (E)	7	6	0.25 mm/12 h	4 and 8 w
Kahraman et al. [16]	2015	Rabbit	New Zealand	Titanium mesh	20	Lower border of the mandible (E)	7	10	0.35 mm/d	45 d
Pripatnanont et al. [17]	2015	Rabbit	New Zealand	Modified Hyrax device, Leone S.p.A., Firenze, Italy	12	Ramus and body of Mandible (E)	3	7	0.5 mm/12 h	4 and 8 w
Kessler et al. [4]	2006	Minipig	Goettingen	Titanium mesh	6	Forehead region	5	10	0.5 mm/d	7, 17, 45 d
Estrada et al. [28]	2007	Rabbit	New Zealand	Titanium plate	12	Forehead region	10	22	0.25 mm/d, 0.5 mm/d	10, 20, 30, 40, 50, 60 d
Lethaus et al. [18]	2010	Minipig	-	Laser-perforated titanium mesh	18	Forehead region	3	5, 10, 15	0.5 mm/12 h	2, 4, 6 w
Sato et al. [19]	2010	Rabbit	New Zealand	Titanium plate	8	Calvaria	7	20	0.5 mm/d	3 w
Tudor et al. [2]	2010	Minipig	Gottingen	Laser-perforated titanium mesh, KLS Martin, Tuttligen, Germany	9	Forhead region	3	5, 10, 15	0.5 mm/12 h	2, 4, 6 w
Zakaria et al. [20]	2012	Rabbit	Japanese	Biodegradable PLLA mesh	8	Calvaria	7	5	0.5 mm/12 h	4, 6 w
Zakaria et al. [21]	2012	Rabbit	Japanese	Titanium mesh	12	Calvaria	7	5	0.5 mm/12 h	4, 6 w
Saulacic et al. [22]	2013	Rat	-	Hemispherical disc	16	Calvaria	7	10	0.4 mm/d	10, 20 d
Saulacic et al. [23]	2013	Rat	Wistar	Titanium plate	48	Calvaria	7	10	0.2 mm/d	7
Saulacic et al. [24]	2016	Rabbit	New Zealand	U-shaped device	60	Calvaria	7	10	0.25 mm/d 0.5 mm/d	10, 17, 24, 31, 77 d
Nakahara et al. [25]	2016	Rat	Wistar	Titanium plate	28	Calvaria	7	10	0.1 mm/d	10 d
Nakahara et al. [26]	2017	Rat	Wistar	Titanium plate	30	Calvaria	7	10	0.1 mm/d	17, 31, 45 d
Zhao et al. [5]	2020	Rabbit	New Zealand	Biodegradable PLLA mesh	18	Calvaria	7	5	0.1 mm/d	8 w

(E): Extraoral; (I): Intraoral; PM: premolar; M: molar; d: days; w: weeks.

**Table 3:** Complications shown in the studies.

Author	Year	Animal Model	Patient Number	Distractor	Complications	No Animals Affected	Period	Major or Minor	Treatment/Outcomes
Schmidt et al. [6]	2002	Rabbit	10	U-shaped body, Synthes Maxillofacial, Paoli, Pa	Lost device	1	Latency	Major	Animal excluded
Sencimen et al. [27]	2007	Rabbit	36	U-shaped device	N	N	N	N	N
Estrada et al. [28]	2007	Dog	4	Titanium plate, Tracper TM Serf. Décines, France	Dehiscence	4	Distraction	Major	Device remove
Casap et al. [29]	2008	Rabbit	10	U-shaped device	Severe infection/Body weight loss > 15%	1 and 1	Latency	Major/Major	Animal excluded
Oda et al. [33]	2009	Rabbit	25	Titanium mesh, M-TAM, Stryker Leibinger, Kalamazoo, MI	Screw loss	1	Consolidation	Major	Animal excluded
Altuğ et al. [30]	2011	Rabbit	36	U-Shaped device	N	N	N	N	N
Bayar et al. [34]	2012	Rabbit	36	U-shaped device	?	?	?	?	?
Inoue et al. [31]	2014	Dog	4	Titanium plate	N	N	N	N	N
Suer et al. [32]	2014	Rabbit	24	U-shaped device	N	N	N	N	N
Kahraman et al. [16]	2015	Rabbit	20	Titanium mesh	N	N	N	N	N
Pripatnanont et al. [17]	2015	Rabbit	12	Modified Hyrax device, Leone S.p.A., Firenze, Italy	Slight Device displacement	2	Consolidation	Minor	Neck collar and conservative
Kessler et al. [4]	2006	Minipig	6	Titanium mesh	N	N	N	N	N
Estrada et al. [28]	2007	Rabbit	12	Titanium plate	Severe infection	12.	Consolidation	Major/Major	Animal excluded
Lethaus et al. [18]	2010	Minipig	9	Laser-perforated titanium mesh	Severe Device displacement	1	Consolidation	Major	Animal excluded
Sato et al. [19]	2010	Rabbit	8	Titanium plate	?	?	?	?	?
Tudor et al. [2]	2010	Minipig	9	Laser-perforated titanium mesh, KLS Martin, Tuttligen, Germany	Slight Device displacement	3	Consolidation	Minor	Conservative
Zakaria et al. [20]	2012	Rabbit	8	Biodegradable PLLA mesh	Mild Infection	2	Latency	Minor	Conservative
Zakaria et al. [21]	2012	Rabbit	12	Titanium mesh	N	N	N	N	N
Saulacic et al. [22]	2013	Rat	16	Hemispherical disc	Post-operative death/Lost device	1 and 1	Surgery/Distraction	Major/Major	Animal excluded
Saulacic et al. [23]	2013	Rat	48	Titanium plate	Severe infection/Lost device	1 and 2	Consolidation	Major/Major	Animal excluded
Saulacic et al. [24]	2016	Rabbit	60	U-shaped device	N	N	N	N	N
Nakahara et al. [25]	2016	Rat	28	Titanium plate	Post-operative death/Lost device	1 and 1	Surgery/Latency	Major/Major	Animal excluded
Nakahara et al. [26]	2017	Rat	30	Titanium plate	N	N	N	N	N
Zhao et al. [5]	2020	Rabbit	18	Biodegradable PLLA mesh	?	?	?	?	?

N: no complications; ?: not reported.

**Table 4:** Quality coefficients of the studies reviewed.

Author	Year	Animal Model	Coefficient	Quality
Schmidt et al. [6]	2002	Rabbit	0.72	Average
Sencimen et al. [27]	2007	Rabbit	0.69	Average
Estrada et al. [28]	2007	Dog	0.66	Average
Casap et al. [29]	2008	Rabbit	0.77	Average
Oda et al. [33]	2009	Rabbit	0.79	Average
Altuğ et al. [30]	2011	Rabbit	0.62	Average
Bayar et al. [34]	2012	Rabbit	0.67	Average
Inoue et al. [31]	2014	Dog	0.67	Average
Suer et al. [32]	2014	Rabbit	0.89	Excellent
Kahraman et al. [16]	2015	Rabbit	0.84	Excellent
Pripatnanont et al. [17]	2015	Rabbit	0.96	Excellent
Kessler et al. [4]	2006	Minipig	0.65	Average
Estrada et al. [28]	2007	Rabbit	0.67	Average
Lethaus et al. [18]	2010	Minipig	0,72	Average
Sato et al. [19]	2010	Rabbit	0.62	Average
Tudor et al. [2]	2010	Minipig	0.79	Average
Zakaria et al. [20]	2012	Rabbit	0.67	Average
Zakaria et al. [21]	2012	Rabbit	0.67	Average
Saulacic et al. [22]	2013	Rat	0.97	Excellent
Saulacic et al. [23]	2013	Rat	0.84	Excellent
Saulacic et al. [24]	2016	Rabbit	0.86	Excellent
Nakahara et al. [25]	2016	Rat	0.89	Excellent
Nakahara et al. [26]	2017	Rat	0.91	Excellent
Zhao et al. [5]	2020	Rabbit	0.86	Excellent

## Supplementary Materials

The following are available online at https://www.mdpi.com/article/10.3390/ani11051233/s1, Table S1: PRISMA Checklist. Table S2: Main results of the studies.
