# Supplementary material for: Systematic Review and Quality Evaluation Using ARRIVE 2.0 Guidelines on Animal Models Used for Periosteal Distraction Osteogenesis
Source: Animals (Basel). 2021 Apr 24;11(5):1233. doi: 10.3390/ani11051233 (PMC8144990; doi:10.3390/ani11051233)
Supplement: Supplementary file 1 [file animals-11-01233-s001.zip › Supplementary Table S2.pdf]

Supplementary Table S2.- Main results of the studies.

Author	Year	Animal	Evaluation Method	QH	BMD (mgHA/mm3)	BV	BV/TV (%)	Tb.Th (µm)	NB Area (mm²)	% NB	Height Gain (mm)	Width (mm)
Schmidt et al. [7]	2002	Rabbit	Histologic and Histomorphometric	Y	-	-	-	-	-	-	2.86	-
Sencimen et al. [29]	2007	Rabbit	Histomorphometric	Y	-	-	-	-	14.4	-	-	-
Estrada et al. [30]	2007	Dog	Radiograph and histologic	Y	-	-	-	-	-	-	-	-
Casap et al. [31]	2008	Rabbit	Micro-CT and Histomorphometric	N	-	13%	41	-	-	-	1.7	-
Oda et al. [32]	2009	Rabbit	Radiographic and histologic	Y	-	-	-	-	19.3	67	-	-
Altuğ et al. [33]	2011	Rabbit	Histologic and Histomorphometric	Y	-	-	-	-	294	-	-	-
Bayar et al. [34]	2012	Rabbit	Histomorphometric	Y	-	-	-	-	16.49	-	-	-
Inoue et al. [16]	2014	Dog	Micro-CT	N	-	-	-	-	3.31	-	-	0.7
Suer et al. [17]	2014	Rabbit	Radiologic, photodensitometric and Histologic	Y	-	-	-	-	-	-	-	-
Kahraman et al. [18]	2015	Rabbit	Radiology, Micro-CT and Histomorphometric	Y	-	62%%	-	0.295	-	-	-	-
Pripatnanont et al. [19]	2015	Rabbit	Micro-CT, Histologic and Histomorphometric	Y	-	53.2%	-	-	-	48	-	-
Kessler et al. [4]	2006	Minipig	Micro-CT and Histologic	Y	-	-	58	-	-	-	-	-
Estrada et al. [30]	2007	Rabbit	Radiograph and histologic	Y	-	-	-	-	-	-	-	-
Lethaus et al. [20]	2010	Minipig	Micro-CT and Histologic	Y	-	-	66	-	-	-	-	-
Sato et al. [21]	2010	Rabbit	Micro-CT, Histologic and Inmunohistochemistry	Y	390	153.7 mm^3	-	-	-	-	6.2	-
Tudor et al. [2]	2010	Minipig	Micro-CT and Histologic	Y	-	-	67	-	-	-	-	-
Zakaria et al. [22]	2012	Rabbit	Micro-CT and Histologic	Y	-	52%	-	-	-	-	-	-
Zakaria et al. [23]	2012	Rabbit	Micro-CT and Histologic	Y		47%	-	-	-	-	-	-
Saulacic et al. [24]	2013	Rat	Histologic and Histomorphometric	Y	109	861 mm^3	-	-	-	-	2.1	-
Saulacic et al. [25]	2013	Rat	Histologic and Histomorphometric	Y	-	-	-	-	-	-	0.79	-
Saulacic et al. [26]	2016	Rabbit	Histologic and Micro-CT	Y	679	35.5 mm^3	-	-	-	-	-	-
Nakahara et al. [27]	2016	Rat	Histologic and Micro-CT	Y	838	23.2 mm^3	-	-	-	-	-	-
Nakahara et al. [28]	2017	Rat	Histologic and Micro-CT	Y	869	12.37 mm^3	-	-	-	-	-	-
Zhao et al. [5]	2020	Rabbit	Histologic and Micro-CT	Y	820	24 mm3	-	-	-	-	-	-

QH: qualitative histology; BMD: bone mineral density; BV: bone volume; TV: tissue volume; Tb.Th: trabecular thickness; NB: new bone; Y: reported; N: not reported
